# Pregnancy after Cytoreductive Surgery and Hyperthermic Intraperitoneal Chemotherapy in Pseudomyxoma Peritonei

**DOI:** 10.1155/2014/694912

**Published:** 2014-06-11

**Authors:** Renato Gomes Campanati, Bernardo Hanan, Sérgio Simões de Souza, Rodrigo Gomes da Silva

**Affiliations:** ^1^Division of Colorectal Surgery, Alfa Institute of Gastroenterology, Hospital das Clínicas, School of Medicine, Universidade Federal de Minas Gerais, Avenida Alfredo Balena 110, 30130-100 Belo Horizonte, MG, Brazil; ^2^Division of Gynecology and Obstetrics, School of Medicine, Federal University of Minas Gerais, Avenida Alfredo Balena 110, 30130-100 Belo Horizonte, MG, Brazil

## Abstract

Pseudomyxoma peritonei is usually a benign tumor that is slightly more common in women. However, it requires aggressive surgical treatment and chemotherapy, often compromising future reproductive function. This report presents a case of pregnancy in a 35-year-old woman who underwent cytoreductive surgery and hyperthermic intraperitoneal chemotherapy for treatment of pseudomyxoma peritonei. The tumor was diagnosed during a laparoscopic examination on a workup for infertility in 2008. Two months later, she underwent a peritonectomy followed by hyperthermic intraperitoneal chemotherapy and, regarding her will to conceive, ovaries and fallopian tubes were preserved. In March 2011, she went through an in vitro fertilization followed by an uneventful pregnancy and delivered a healthy child 39 weeks later. Ovaries are usually resected during the cytoreductive surgery, since they are common sites for neoplastic implants, and, even when not performed, little is known about the effects of local chemotherapy on female fertility. The largest international survey on this matter only describes seven similar cases. This particular report not only describes a rare condition but also adds to the upcoming discussion about whether ovaries can be preserved in specific situations, therefore keeping the prospect of conceiving after this treatment.

## 1. Introduction

Pseudomyxoma peritonei (PMP) is a rare clinical condition characterized by mucinous ascites due to the growth of a tumor on the peritoneal surface. PMP usually develops from primary appendiceal epithelial neoplasms, which leads to its own lumen obstruction and subsequent perforation. Subsequently, the neoplastic cells colonize the peritoneal cavity and begin to produce a great amount of mucin [1, 2]. The appendix is the most common primary tumor site (from 52% up to 91% of cases), but it has also been described after ovarian, colorectal, and pancreatic tumors [1–3].

PMP's incidence is reported as being 1 per 1.000.000 people annually, is more commonly diagnosed in women, and has a mean diagnostic age of 52 years [1, 2]. About 75% of all appendiceal tumors are noninvasive and, therefore, grow slowly and allow patients to survive for decades. However, it can also develop as fast growing invasive tumors, which can lead to death 1 to 2 years after initial diagnosis [1, 4].

The classical PMP treatment is surgical debulking of the tumor in order to alleviate the effects of abdominal pressure. It is a palliative treatment and has an increased probability of leaving the remaining neoplastic cells in the peritoneal cavity. Subsequent surgeries may be necessary and are much more difficult due to intra-abdominal adhesions [1]. Nevertheless, a more effective treatment was described by Sugarbaker and consists of cytoreductive surgery (CRS) combined with hyperthermic intraperitoneal chemotherapy (HIPEC) [5]. The aim of this strategy is to resect visible disease and eliminate any occult neoplastic cells with a high concentration of chemotherapy drugs at 41-42 degrees Celsius. This approach has achieved better results in comparison to debulking surgery alone from historical control. For instance, a multicenter study conducted by Elias et al. demonstrated a 5-year overall survival rate of 73% and a disease-free survival rate of 56% [1]. In our colorectal unit, within 30 patients who underwent CRS and intraperitoneal chemotherapy in a period of 9 years, we observed a 5-year global survival of 77% (unpublished observations).

Although the median age of diagnosis is around 50 years, some young patients in fertile age present with PMP and maintain the desire to conceive. The pelvic peritonectomy generally involves the resection of both ovaries, since these organs are common sites of occurrence and recurrence of peritoneal seeding. Therefore, the preservation of the ovaries in this setting is controversial. With the current perspective of an increasing life expectancy after PMP surgery and the known effects of chemotherapy on the female reproductive system, much has been questioned about the fertility of these women and the prognosis of pregnancy after CRS and HIPEC. The largest international study on this matter has reported only seven cases of successful pregnancies after the treatment suggested by Sugarbaker [6].

We report here the case of a 35-year-old woman diagnosed with PMP and submitted to CRS and HIPEC that conceived a healthy child 3 years after surgery.

## 2. Case Presentation

On August, 2006, a 35-year-old nulliparous female presented to her gynecologist for evaluation of infertility due to polycystic ovary syndrome, which was confirmed by clinical findings and laboratory tests. At that time, three ovulation induction cycles with clomiphene citrate and gonadotropins were performed with no success.

On October, 2007, she went through another three ovulation induction cycles along with intrauterine insemination. Although ovulation was confirmed by ultrasound examination, no embryos were detected.

Due to failure on later treatments, she was submitted to a laparoscopic examination in May, 2008, which demonstrated several peritoneal implants of neoplastic cells. Biopsy showed low-grade mucinous neoplastic cells, which it is a diagnostic compatible with PMP.

The patient was completely asymptomatic and no abdominal masses or ascites were found on clinical examination. All laboratory tests were normal (Hb: 13,6 mg/dL; Ht: 40,1%; fasting glucose: 82 mg/dL; creatinine: 0,7 mg/dL; HBsAg: negative; TSH: 1,76 mg/dL) and a hysterosalpingography showed pervious fallopian tubes.

On July, 2008, after evaluation at our colorectal unit, the patient underwent an exploratory laparotomy in order to perform CRS and HIPEC. The appendix was the primary tumor site and there was no macroscopic disease in both ovaries, enabling their preservation. Peritonectomy of the right subphrenic space, greater omentectomy, and appendicectomy were performed, followed by hyperthermic intraperitoneal chemotherapy with mitomycin C (25 mg) at 41°C over 90 minutes. The completeness of cancer resection (CCR) was classified as CCR-0, meaning no macroscopic neoplastic implants, and the patient's recovery was uneventful.

The histological examination showed a disseminated peritoneal adenomucinosis (DPAM) type of appendiceal PMP, according to Ronnett's classification [7], on the right subphrenic peritoneum, greater omentum, cecal appendix, and liver capsule.

The patient remained asymptomatic, with only one sporadic episode of acute abdominal colicky pain and diarrhea. Subsequent magnetic resonance and ultrasound imaging of the abdominal cavity were done the following 5 years and no recurrences or ascites were found.

At 39 years of age, on September, 2010, she decided on in vitro fertilization and three embryos were implanted on March, 2011, with positive beta HCG twelve days later.

She had an uneventful pregnancy and delivered from caesarean section a healthy female fetus on the 39th week, on November, 2011. Fifteen days after the delivery, she presented with a hypertensive crisis and was clinically treated with good response.

## 3. Discussion

The introduction of CRS and HIPEC for the treatment of PMP has substantially increased overall and disease-free survival rates, turning it into the gold standard treatment not only for PMP specifically, but for other types of peritoneal carcinomatosis (PC) as well [1, 8, 9]. Usually, the ovaries are resected in this procedure, since they are a common implantation site of neoplastic cells. Moreover, PC presents itself as a poor prognostic factor for future pregnancies.

Nevertheless, recent evidence depicts the possibility of ovary preservation and childbearing after CRS and HIPEC [6]. Elias et al. questioned the systematic removal of the ovaries during PC surgery, which was based on a single study where only 18 out of 194 women were less than 41 years old. A new policy was proposed concerning the decision of whether or not to resect ovaries, taking into account neoplastic cell implants (macroscopic implants or clinical suspicion) and the patient's wishes regarding future pregnancy. With these criteria applied, ovarian preservation was possible on 44% of patients and tumor recurrence was detected in only 14% of them [10].

Additionally, an international survey taken among the International Peritoneal Surface Malignancy Group was able to detect seven pregnancies in women who have undergone CRS and HIPEC. The women's ages varied from 17 to 34 years and 5 of them had the diagnosis of PMP. The most frequent delivery was vaginal, all of them within 2 years after the procedure, and only 1 newborn had a congenital problem (diaphragmatic hernia) [6].

This case report, along with the data presented, shows that pregnancy after CRS and HIPEC is a possibility. Our patient conceived only after in vitro fertilization because she had a previous fertility problem, but there was no alteration of her reproductive function.

The PMP diagnosis was made during the workup for infertility, such as one of the cases in the Ortega-Deballon et al. study [6]. Recently, finding this condition in younger patients is becoming more frequent, which is bringing up questions on its impairment of fertility and the need to take into account the woman's wishes regarding conceiving when planning the CRS.

In conclusion, there is plenty of consistent evidence showing that CRS and HIPEC are the gold standard approach for PMP and that pregnancy in fertile female patients who underwent these procedures is possible. In addition, preservation of ovaries and adnexa may be considered among these women based on specific criteria [10]. Finally, reproductive assisting techniques, such as in vitro fertilization, can be used to increase the PMP patients' probability of conceiving. Furthermore, the surgical oncologist must work together with the fertility specialist on the decision making process.
